# Neuromotor Regulation of Ankle Stiffness is Comparable to Regulation of Joint Position and Torque at Moderate Levels

**DOI:** 10.1038/s41598-020-67135-x

**Published:** 2020-06-25

**Authors:** Alexander M. Wind, Elliott J. Rouse

**Affiliations:** 1 Neurobionics Lab, University of Michigan, Ann Arbor, MI 48109 USA; 2 Department of Biomedical Engineering, Northwestern University, Evanston, IL 60208 USA; 3 Shirley Ryan AbilityLab, Chicago, IL 60611 USA; 4 Department of Mechanical Engineering, University of Michigan, Ann Arbor, USA; 5 Robotics Institute, University of Michigan, Ann Arbor, USA

**Keywords:** Biomedical engineering, Mechanical engineering

## Abstract

Joint mechanical impedance, which describes the instantaneous relationship between kinematic perturbations and the resulting torque response, plays an important role in the way humans ambulate, interact with the environment, and respond to disturbances. Recent studies have quantified how the stiffness component of mechanical impedance varies during walking. However, the extent to which humans can voluntarily regulate leg joint stiffness is not yet known. Our study sought to quantify the accuracy and precision of the neuromotor system to voluntarily regulate ankle joint stiffness while seated, and compare these data to the well-known abilities to regulate ankle joint torque and position. We tested individuals’ ability to to regulate these quantities at three different magnitudes: 20%, 40%, and 60% of a maximum value. Our results showed that subjects were able to voluntarily regulate ankle joint stiffness, and that the normalized accuracy and precision of stiffness regulation were not different than those of position or torque for targets at magnitudes of 20% of a maximum value. However, the accuracy and precision of stiffness regulation were statistically different than those of position and torque for targets at magnitudes of 40% of the maximum values. At moderate targets, the similarity of the ability to regulate ankle joint stiffness when compared to the abilities to regulate joint torque and position highlights the importance of a comprehensive description of lower-limb biomechanics that includes consideration of joint mechanical impedance, in addition to the common descriptions of joint torque and position.

## Introduction

Our fundamental understanding of how human locomotion is regulated by the neuromuscular system mainly stems from analyses of how the legs are moved during gait (joint angles), and how effort is provided throughout locomotion (joint torques)^1^. These data have formed the core of locomotor biomechanics for over a century. In addition, this information has many important implications, including serving as a benchmark for diagnosis and treatment of neuromotor pathologies and providing a guide for the development of assistive technologies, among other uses. However, this fundamental biomechanical description of human locomotion is incomplete.

The human neuromuscular system permits regulation of joint stiffness properties independent of joint torque and angle. When contracted, muscles only provide force in tension—they cannot provide forces in compression. Consequently, bidirectional joint torques are achieved using agonist-antagonist muscle groups. The simultaneous co-activation of agonist-antagonist muscles (among other mechanisms)^2^ increases the mechanical stiffness of a joint, without necessarily affecting the angle or net torque^3,4^. Joint stiffness, along with inertial and viscous properties, comprise a representation of *mechanical impedance*, components of which that can be varied throughout movement^4–7^. Joint mechanical impedance regulates the instantaneous torque response that accompanies changes in joint position, governs the body’s response to unexpected disturbances, and regulates how energy is stored, exchanged, and dissipated by the joints of the leg^8^—concepts critical to human locomotion^9^. Thus, mechanical impedance is a fundamental biomechanical property that varies continuously and subconsciously during gait, alongside joint angles and torques.

Much is known about leg joint mechanical impedance during static, postural tasks. Mathematically, impedance is typically represented as a second order system (stiffness, damping, and inertia)^3,10^, and is estimated using system identification analyses. During static tasks, the influence of many factors on joint impedance has been characterized^11–21^. Historically, these properties have been difficult to measure during dynamic tasks, such as locomotion. However, recently, several groups have pioneered the tools to quantify how ankle impedance varies during locomotion. Rouse *et al*. showed that the stiffness component of ankle impedance increases throughout the stance phase of walking^22,23^, and Shorter and Rouse demonstrated that ankle stiffness decreases during the terminal stance phase^24^; Lee and Hogan showed that stiffness remains low during swing phase of gait, increasing slightly before the onset of the subsequent stance phase^23,25^. Combined, the results of these studies have provided a more comprehensive understanding of gait biomechanics, complementing the traditional biomechanical gait representations of joint angle and torque variation.

Mechanical impedance has provided new insight into locomotor biomechanics, but little is known about how the nervous system regulates these quantities. Discovering how humans can voluntarily regulate their joint mechanical impedance is an important step in expanding our modern understanding of human locomotor control. Researchers have previously studied the accuracy and precision of the control of ankle joint position and torque, elucidating key properties that describe the human ability to regulate biomechanics. Deshpande *et al*. and others showed that humans can achieve an ankle joint position accuracy of approximately 2 degrees, approximately 3% of the population average range of motion, with a standard deviation (*i.e*. precision) of 1 degree^26–29^; the high accuracy and precision of ankle position regulation highlights its importance in neuromotor control. In addition, researchers have quantified humans’ ability to regulate ankle torque, and have shown that people are able to reproduce both plantar flexion and dorsiflexion torques within an average of 1.7 N·m ± 1.2 N·m, approximately 5% of the population average maximum voluntary contraction in either direction^29^. Although this previous work has provided much insight into the capacity of the neuromotor to closely regulate ankle joint position and torque, it cannot be used to infer information about regulation of ankle stiffness. Researchers have recently investigated how humans *perceive* changes in joint stiffness. Azocar and Rouse have determined that humans are able to detect a 12% and 13% change in stiffness, when interacting with a virtual mechanical impedance at the ankle and knee, respectively^30^. Elucidating sensorimotor perception is one important step towards fully characterizing the proprioception and neuromotor regulation of joint impedance, but the accuracy and precision of the individual’s ability to voluntarily regulate joint stiffness remains unknown.

The objective of this study was to quantify the accuracy and precision of the human ability to regulate ankle joint stiffness, and to compare these data to the more commonly-studied regulation of joint torque and position. We chose to study the stiffness component of mechanical impedance because it changes significantly during gait, and accounts for the vast majority of the torque response^22,24^. We focused on the ankle joint because of its importance to locomotion, adding the majority of mechanical energy used to propel the body forward^1^. The intent of this work is to provide insight into how individuals voluntarily regulate ankle joint stiffness, and to quantify previously-unknown fundamental information about human neuromotor control. We hypothesized that there would be significant differences in the accuracy and precision of an individual’s ability to match a specified target stiffness (e.g. accuracy at the 20% target value would be different than accuracy at the 40% target). We additionally hypothesized that there would be a difference in accuracy and precision of target matching when comparing ankle stiffness matching to ankle torque and position matching tasks. Our results demonstrated that the accuracy and precision of ankle stiffness control was not statistically different than the accuracy and precision of ankle torque or position control in matching tasks. Specifically, we found that performance across the three domains (*i.e*. impedance, position, and torque) was most similar at moderate intensity levels, with stiffness matching error increasing with higher muscle activation.

## Results

**Figure 1:**
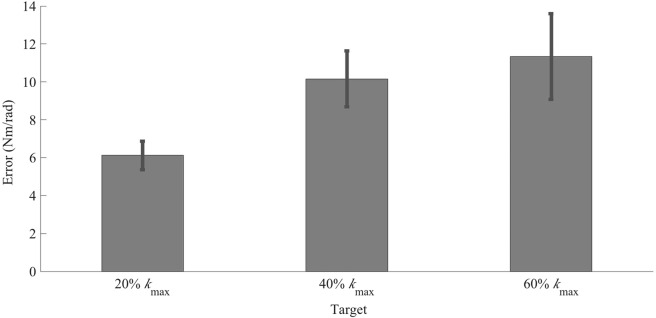
Comparison of absolute value of error (*i.e.*, accuracy) averaged across subjects for each target level. Across subjects, 20% of *k*_*max*_ ranged between 26.0–44.6·N m/rad, 40% of *k*_*max*_ ranged between 40.8–54.7 N·m/rad, and 60% of *k*_*max*_ ranged between 55.5–64.8·N m/rad. *Denotes a statistically significant difference in accuracy between two target levels. Error bars denote standard error of the mean which, while proportional to standard deviation, do not directly represent precision in this figure.

In this study, subjects attempted to match their ankle joint stiffness to a desired target value. To modulate joint stiffness, subjects co-contracted agonist-antagonist muscles of the ankle, while maintaining minimal net torque. The target values were 20%, 40%, and 60% of a maximum stiffness value (*k*_*max*_) determined in a pilot study. For each stiffness target, 14 subjects were permitted 12 sequential attempts to match the target value, with accuracy feedback provided at the end of each attempt. Analyses were performed on the final 10 trials of each task to mitigate the effect of discovering target location in the first two trials. Our results show that, following a short training protocol, subjects were able to modulate stiffness via voluntary isometric co-contraction of the muscles about the ankle. Mean absolute error ranged from approximately 6.1 N·m/rad to 11.3 N·m/rad, with a precision of approximately 2.8 N·m/rad to 8.5 N·m/rad. Averaging across all subjects and all target levels, the average error in stiffness target matching was 9.2 6.3 N·m/rad (mean s.d.). To compare target matching accuracy (mean of error) and precision (standard deviation of error) across target magnitudes, a statistical analysis of variance (ANOVA) was performed. Accuracy varied significantly between target magnitudes (*F*_2,41_ = 5.89; *p* = 0.008) and subjects

(F_13,41_ = 4.19; *p* < 0.001). Similarly, precision differed significantly between target magnitudes (*F*_2,41_ = 5.28; *p* = 0.01) and subjects (*F*_13,41_ = 3.76; *p* = 0.002) (Fig. 1). A post-hoc comparison showed that the mean accuracy for the 20% target was different than the mean accuracy of the 60% target (*p* = 0.05) and the mean precision of the 20% target was significantly different than the mean precision of the 40% target (*p* = 0.01).

We conducted an analogous experiment of matching joint position or torque to provide a comparison between regulation of joint stiffness, each described as a different *domain*. Subjects attempted to match their ankle joint angle or torque to desired target values which were functions of maximum reachable plantar flexion angle (***θ***_*max*_) and maximum reachable plantar flexion torque (***T***_*max*_), respectively. Analogous to the stiffness matching tasks, accuracy feedback was provided following each trial, and analyses were performed on the last 10 trials.

**Figure 2:**
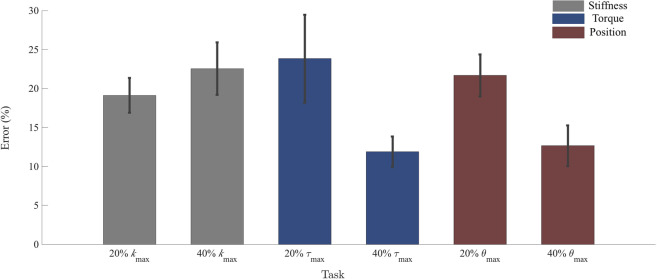
Percent error across task domains, averaged across subjects. Targets were a function of maximum joint stiffness (*k*_*max*_), maximum voluntary plantar flexion torque (***T***_*max*_), and maximum reachable plantar flexion angle (***θ***_*max*_), for tasks of stiffness, torque, or position, respectively. Neither target matching accuracy nor precision were shown to vary significantly between domains. Error bars denote standard error of the mean which, while proportional to standard deviation, do not directly represent precision in this figure.

The inter-subject average percent error for all tasks was used to compare accuracy and precision between position, torque, and stiffness target matching abilities (Fig. 2). An ANOVA was performed using domain and target magnitude as fixed factors, and subject as a random factor. Target matching accuracy did not differ significantly between the different domains (*F*_2,83_ = 0.82; *p* = 0.45), nor was there a significant difference between subjects (*F*_13,83_ = 2.34; *p* = 0.11). However, accuracy did vary significantly between target magnitudes (*F*_1,83_ = 6.0; *p* = 0.03), and there was a significant interaction between domain and target magnitude (*F*_2,83_ = 4.19; *p* = 0.03). Differences in precision with respect to domain approached, but did not achieve, significance (*F*_2,83_ = 3.11; *p* = 0.06). Precision also did not differ significantly between subjects (*F*_13,83_ = 2.41; *p* = 0.14). However, differences in precision between target magnitudes were significant(*F*_1,83_ = 7.0; *p* = 0.02) and the interaction between domain and target magnitude also approached significance (*F*_2,83_ = 3.11; *p* = 0.06).

Three targets were collected for each domain; however, the torque and position domain included one target in the dorsiflexion and two targets in plantar flexion direction. These targets were chosen because both positive and negative values have meaning in the position and torque domains. Stiffness, however, is only physically meaningful for positive values–the analogy between the domains does not hold for this concept. For position and torque, data were collected for one dorsiflexion target at the 20% of maximum level, and these data were compared to the 20% plantar flexion target. There was no statistical difference between plantar flexion and dorsiflexion, thus analyses focused on the plantar flexion direction only (torque: *F*_1,27_ = 0.34, *p* = 0.57; position: *F*_1,27_ = 2.17, *p* = 0.16). Finally, to facilitate direct comparison, statistical tests across domains were performed for targets that were achieved in all tests (20% and 40% of the domains’ maximum levels).

In this study, stiffness was estimated using a system identification algorithm, and Variance Accounted For (VAF) was used to estimate model fit. VAF quantified what percentage of the variance of the measured torque data was explained by the estimated torque data, where estimated torque was obtained via the mechanical impedance model. High VAF values were obtained for all stiffness trials (mean VAF = 97.3 ± 2.4), demonstrating that the mechanical impedance model fit the data well.

## Discussion

Until recently, the mechanics of locomotion were described mainly by the regulation of joint position and torque; however, new studies have quantified the underlying mechanical impedance of the ankle, which also varies during gait^22–24^. The stiffness component of mechanical impedance is an important property for locomotion, since it governs the body’s response to irregularities in terrain, as well as how mechanical energy is stored and transferred between the joints of the legs. However, despite the recent quantification of these properties during gait, a gap remains in our understanding of how the neuromotor system regulates leg joint impedance. The intent of this study was to provide insight into the human ability to voluntarily regulate ankle joint stiffness, specifically in terms of accuracy and precision. Co-activation of antagonist muscles has previously been shown to enable voluntarily modulation of joint stiffness during static postural conditions^4^, but, to the best of our knowledge, this strategy had not been quantitatively measured in healthy subjects, or examined in the context of the larger framework of motor control. Consequently, in this study we compared the ability to regulate ankle joint stiffness to the well-known and intuitive abilities to control joint position and torque. These data lay the foundation for understanding the importance and limitations of the neuromotor system’s ability to voluntarily regulate leg joint dynamics.

Subjects were able to voluntarily regulate ankle joint stiffness to match targets that spanned achievable joint stiffness magnitudes. Target matching accuracy and precision decreased with increasing target magnitude. The stiffness magnitudes chosen in this study were based on the maximum achievable ankle stiffness obtainable during isometric co-contraction, with near-zero net torque. However, ankle stiffness in this configuration is substantially less than what is observed at different ankle torques and joint angles, such as what has been shown during locomotion. For example, during gait, ankle stiffness is as high as approximately 420·N m/rad for a 70 kg individual, almost ten times greater than what was tested in this study^22–24^. However, the high stiffness during locomotion is likely the result of approaching the ankle joint’s range of motion limits, and thus is not due to muscle activation alone, and is compounded by the parallel stiffness of the physiological mechanical structures^19^. Future work is needed to ascertain how joint stiffness regulation is affected by covariation of net torque and joint angle outside of the neutral configuration.

Accuracy and precision compared across domains were not significantly different at the lower target magnitude, but differed significantly at higher magnitude. For the target at 20% of the domain’s maximum, accuracy and precision were comparable across all domains (Fig. 2). However, when evaluated across domains at the 40% target, accuracy and precision of the torque and position domains were greater than what was obtained for joint stiffness, noted by the interaction terms in the statistical model. The increase in both accuracy and precision for position and torque at higher targets occurred because the mean and standard deviation of absolute error in those domains remained relatively constant as target magnitude increased, thereby reducing percent error at larger target magnitudes. This consistency across target levels in position and torque was also reported by Deshpande *et al*.^29^. The near constant error may be the result of the limits of physiological sensing mechanisms, such as Golgi Tendon Organs that sense muscle force, or muscle spindles and mechanoreceptors that assist in the sensation of joint angle^31^. In contrast, both the accuracy and precision of stiffness error (absolute value) increased with increasing target magnitude. The opposing trends may result from the unfamiliar nature of stiffness regulation, when compared to the intuitive concepts of angle and torque regulation. All subjects were given time to become familiar with the task of co-contraction to increase joint stiffness and were able to achieve the targets. However, longer training periods may improve stiffness accuracy within the limited number of trials assessed in this work. Alternatively, the reduction in stiffness domain accuracy and precision may result from the lack of direct physiological sensation of stiffness, which instead must be indirectly sensed via fusion of joint torque and position sensations. It is also important to note that while both results are reported and discussed here, the accuracy results cannot be generalized to longer analysis windows, as accuracy effectively loses meaning as the number of trials tends towards infinity. Further studies are needed to determine how target-matching accuracy and precision may change with greater training and skill.

Our results are consistent with previous studies investigating the ability to regulate ankle joint position and torque. While voluntary control of endpoint stiffness has been studied in the upper limb^32^, there are no known studies investigating the regulation of ankle stiffness through co-contraction of antagonist muscle pairs. Deshpande *et al*. reported that the absolute error of torque and position reproduction were measured to be approximately 1.7·N m and 2.3° respectively^29^. In their study, the target torques were 10·N m in both directions, and the target positions were 5° plantar flexion, 10° plantar flexion, and 5° dorsiflexion. In our experiment, the 20% torque target level was approximately 10·N m in both plantar flexion and dorsiflexion for all subjects. At these levels, we measured absolute error of torque reproduction to be 1.6 N·m. Similarly, our target levels for position were approximately 5° and 10° plantar flexion and 5° dorsiflexion. Averaging across the three, we measured an average error of 1.1°. These results demonstrate a high degree of consistency between our current work and previous similar studies.

An improved understanding of how the neuromotor system voluntarily regulates joint stiffness may aid the development of better and more biomimetic robotic prostheses, exoskeletons, and humanoid robots. The design and control of many recent technologies is motivated by mechanical impedance, and many current devices incorporate impedance control based on the human body^33–39^. Impedance control is often chosen because it enables compliance between the machine and environment. Although impedance control is implemented in these systems, the specific impedance parameters, including joint stiffness, are not based on physiologically realistic values, as these data have not been available until recently. Instead, the mechanical impedance parameters are typically determined ad hoc or heuristically^35,36,40–46^. Modeling the impedance characteristics of these technologies based on the human body has several potential advantages, including an improved ability to react to unexpected mechanical perturbations arising from missteps or slips during ambulation. Thus, to develop biomimetic wearable robotic systems and humanoids, future consideration should be given to the human ability to regulate joint stiffness and how this knowledge can inform our robotic control policies.

This study was performed under static conditions, and the generalizability of our results to dynamic tasks, such as locomotion, is not yet clear. The static experimental setup, while capable of providing preliminary insight into the ability to regulate stiffness via isometric co-contraction, is not directly analogous to locomotion, when position, torque, and muscle activation are changing simultaneously. However, due to the experimental challenges associated with measuring stiffness regulation during dynamic tasks, our initial investigation focused on estimation during isometric conditions. In addition, this work used a neutral ankle angle and zero net torque (for the stiffness and position domains). It is likely that the ability to control stiffness, position and torque may depend on the configuration of the limb. Future experiments are needed to ascertain how stiffness can be regulated in dynamic tasks and at varying operating conditions.

Our goal was to quantify the neuromotor ability to voluntarily regulate joint stiffness. As voluntary regulation of joint stiffness via muscular co-contraction is a novel task for most people, learning to co-activate muscles may have affected the results. We attempted to minimize the influence of motor learning on our results through several measures. First, the order of domains was randomized, as was the order of targets within a domain, for each subject. This randomization served to minimize the effects of any confounding factors that may co-vary with task order. Additionally, all subjects performed practice co-contraction trials preceding the stiffness protocol. These were included to confirm all subjects were proficient in maintaining equal co-contraction throughout perturbation trials, which was necessary to assure our measurements were representative of physiological stiffness. Subjects completed practice trials until they could complete three consecutive trials with net torque sufficiently close to zero, as indicated by the trials each having a Variance Accounted For (VAF) above 90%, which confirmed our mechanical impedance estimation model fit the data well. Trials with VAFs above 90% had low variability in estimated stiffness and were consistently achievable by subjects. Finally, only the last 10 of the 12 trials were included in our data analysis to eliminate the period at the beginning of each trial in which subjects were attempting to locate the target.

Joint stiffness is known to depend on multiple factors, and while our objective was to isolate voluntary changes in joint stiffness, these other factors may have influenced our results. Stretch reflexes, passive tissue properties, and muscle activation all contribute to physiological joint stiffness, and our experiment was designed to mitigate the effects of reflexes and passive tissue properties. The stiffness from passive tissue properties was measured and subtracted from the estimated stiffness values. Reflex contributions can be minimized by carefully selecting the parameters of the mechanical perturbations applied to the ankle during stiffness measurements. Changes to joint position elicits monosynaptic reflex responses, which can impact joint dynamics^47,48^. Voluntary prediction and phase locking, which dominate reflex dynamics, were minimized by selecting stochastic low-frequency perturbations^3^. Despite these measures, it is possible that intrinsic reflex responses contributed to the measured stiffness values.

The maximum values used to scale target locations were inconsistent across domains. Torque and position targets were scaled based on subject-specific ranges; however, a single value for maximum stiffness was used for all subjects, which was determined via a pilot study. Our decision to use a single value for maximum stiffness stemmed from the difficultly and highly fatiguing nature of determining subjects’ maximum voluntary stiffness. Furthermore, some subjects required many more trials to obtain usable maximum stiffness values, which created inconsistency in the training protocol. Using a constant value for maximum stiffness across subjects may have increased the variability in the accuracy and precision but would not have affected the mean values obtained.

## Methods

### Subjects

Fourteen able-bodied subjects (7 men, 7 women) aged between 20 and 51 years (26.8 10.2 years; mean s.d.) were recruited to participate in the study. Subjects had no prior history of neurological disease or injury to the ankle, and informed consent was obtained prior to beginning the experiment. The protocol was approved by the Institutional Review Board of Northwestern University (IRB protocol STU00202024), and all research was performed in accordance with relevant guidelines and regulations.

### Equipment

**Figure 3:**
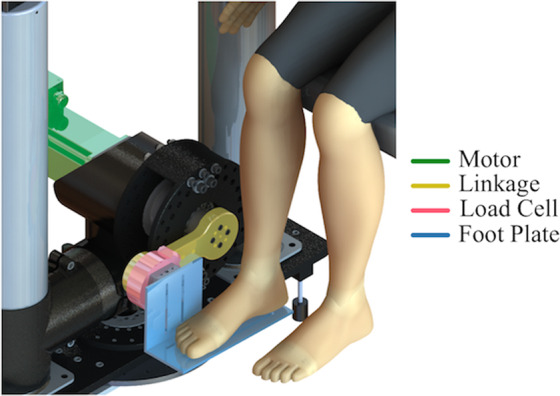
Experimental setup showing the rotary dynamometer and relevant components. Subjects interacted directly with the foot-plate (blue) which rendered the various experimental conditions. Position was measured by the encoder, torque was measured by the load cell (red), and mechanical impedance was calculated from the position and torque data using system identification techniques. The subject’s foot was placed in a rigid plastic orthosis (not shown), and their ankle center of rotation was aligned to the axis of rotation of the dynamometer.

Subjects were seated in an adjustable chair (model: 835-000, Biodex Medical Systems, NY) coupled to the Neurobionics Rotary Dynamometer a custom frame-mounted motor (model: BSM90N-3150AF, Baldor, Fort Smith, AR, USA) with a six-axis load cell (model: 45E15A4, JR3, Inc., Woodland, CA, USA) (Fig. 3) controlled in real time using MATLAB xPC (Natick, MA, USA). The chair was adjusted such that the relative angles between the thigh and shank (knee angle), and the shank and foot (ankle angle) were both at 90°. All angles measured throughout the experiment were measured as deviations from this neutral position. The right ankle was secured rigidly to a plate fixed to the dynamometer output shaft via a form-fitting, rigid plastic orthosis. The axis of rotation of the dynamometer was aligned with the center of rotation of the ankle with a concentric laser. Movement of the shank was minimized and the knee angle was kept at 90° by straps secured across the mid-thigh. Standard preparation preceded the affixation of four bipolar surface electrodes to record surface electromyographic (EMG) activity of the tibialis anterior, lateral gastrocnemius, medial gastrocnemius, and soleus (model: Bagnoli, Delsys, Boston, MA, USA). EMG data were collected but are not presented as part of this study.

Their foot was secured to the foot-plate via Velcro and a set of straps across the top of the foot and ankle joint. The seat was adjusted such that the ankle joint and knee joint were both at 90 degrees and the thigh was then secured in place with straps. Figure adapted from Azocar 2017^30^.

### Protocol

#### Overview

**Figure 4:**
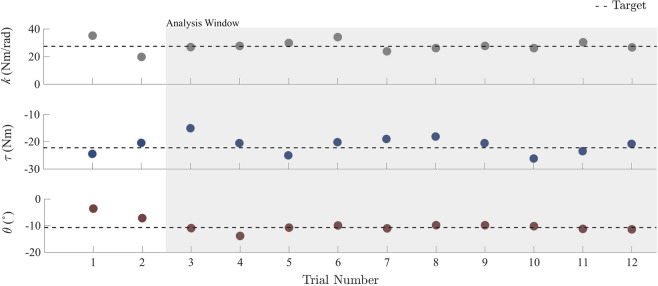
Representative plots of completed target-matching tasks. Each point on the plot represents the average magnitude of either stiffness, torque, or position produced by the subject during a trial. Torques and angles in the plantar flexion direction have negative magnitude by convention. During the experiment, the plot begins with no data, and is updated with a new data point following each trial. In each trial, the subject attempts to match the point as close as possible to the target (dotted reference line). The subject was able to adjust his or her own performance based on the results of the preceding trials.

The complete experimental protocol consisted of nine target-matching tasks, divided into three domains – stiffness, torque, and position – each with three targets. The number of targets was chosen to balance subject fatigue and experimental length. The subjects were given 12 trials per task, where subjects attempted to match their ankle joint stiffness, torque, or position to a target value in the corresponding domain, moving from an initial value of zero for all domains. Subjects were given no live feedback during a trial and could only observe their performance following the completion of each trial. This paradigm is analogous to playing darts; each task was repeatedly performed, permitting the subject to make corrections between each trial, based on the performance observed in the preceding trial. Subjects were provided with a graphical display of their performance throughout the task, where the x-axis enumerated trial number and the y-axis displayed the magnitude of stiffness, torque, or position, with a horizontal line corresponding to the target value. Immediately following the completion of each trial, the subject’s performance was updated on the plot. This display allowed subjects to visualize whether they had overshot, undershot, or correctly matched the target value (Fig. 4). Accuracy was quantified by the mean error from the target, and precision was determined using the standard deviation across trials for a given task. The order of domains, as well as the order of targets within a domain, were randomized. All three target-matching tasks within a domain were completed before moving on to the next set of three tasks.

#### Stiffness protocol

To measure accuracy and precision of joint stiffness regulation, subjects attempted to match their ankle stiffness to a target value via isometric co-contraction of the muscles of the ankle. Stiffness was measured by applying system identification techniques to torque and position data collected by the dynamometer. During each trial, Gaussian white noise perturbations with a mean amplitude of 2° were filtered using a 4th order Butterworth filter with a cutoff frequency of 5 Hz, and applied using the dynamometer. Prior to the experiment, three estimates of resting ankle joint stiffness were obtained where the subject did not co-contract. These data were used to measure the stiffness generated purely by passive musculotendon properties. Targets were subsequently calculated to be 20%, 40%, and 60% between the subject’s passive stiffness and a maximum stiffness value obtained in a pilot study prior to the experiment. This maximum stiffness value was calculated from the pilot study in which nine able-bodied subjects (5 male, 4 female) were instructed to exert a maximum co-contraction of the muscles about their ankle during 10-second duration perturbation trials. The maximum stiffness for our experiment was chosen to be 85·N m/rad, which was the highest stiffness reachable by all subjects in the pilot study. The mean maximum stiffness across all subjects in the pilot study was 92.3·N m/rad with a standard deviation of 26.6·N m/rad. Full data from the pilot study is available in Supplementary Table 4. Due to the non-intuitive nature of achieving a desired joint stiffness, subjects completed a training protocol in which they practiced co-contracting the muscles that span the ankle to obtain a desired joint stiffness. All stiffness values were calculated from perturbation trial data using an existing system identification algorithm^10^. Briefly, the algorithm estimated an impedance impulse response function from the average of a time-varying and time-invariant cross-correlation function (*φ*_*xy*_) and the pseudoinverse auto-correlation matrix (*φ*_*xx*_). This impedance impulse response function was subsequently integrated over time to yield an estimate of joint stiffness, the static component of the mechanical impedance transfer function. Stiffness was calculated from the middle eight seconds of the analysis window, with one second at the beginning and end of each trial removed to eliminate any artifacts arising from starting and stopping the trial. The stiffness value calculated from each trial was then used for analysis and display, and the protocol was repeated for each of the three stiffness targets.

#### Torque protocol

For a comparison with the neuromotor ability to voluntarily regulate joint torque, we quantified accuracy and precision of ankle torque regulation. Accuracy and precision of ankle joint torque were directly measured by the dynamometer. Isometric maximum voluntary contractions (MVCs) were collected prior to the target-matching protocol in both the plantar flexion and dorsiflexion directions. Targets were calculated to be 20% of the subject’s plantar flexion MVC, 40% plantar flexion MVC, and 20% dorsiflexion MVC. Similarly to the stiffness protocol, the number of targets was chosen to prevent fatigue, while adequately spanning the range of joint torques. In each trial, the dynamometer held a fixed position of 0° while the subject attempted to match the torque target. Torque data were recorded throughout the trial, and the middle three seconds were used for analysis and display. One second at the beginning and end of each trial were removed to eliminate any effect of the subject starting and stopping the trial. The average change in torque during the analysis window was 4.6·N m/rad. The protocol was repeated for each of the three torque targets.

#### Position protocol

For a comparison with the neuromotor ability to control joint position, we quantified accuracy and precision of ankle position regulation. In this task, subjects attempted to match their ankle joint position to various target positions, measured as excursions from the initial configuration detailed above. All positions reported herein are relative to this initial configuration. We measured the ability to match excursion angle, rather than an absolute position, to avoid inconsistencies that arise when defining a reference equilibrium angle (*i.e. θ* = 0) for use in calculating percent error. Initially, the subject’s range of motion was quantified. Targets were chosen to be 20% of the maximum plantar flexion angle, 40% of the maximum plantar flexion angle, and 20% of the maximum dorsiflexion angle. In each trial, the subjects interacted with the dynamometer with admittance control, which rendered the dynamics of a virtual spring-damper system and allowed the subject to freely move his or her ankle. The stiffness component of the virtual spring mass damper was selected to be 0.1·N m/rad, the damping component 20·N m s/rad, and the inertia 1 kg·m^2^. The mechanics of the spring-mass-damper were chosen so that there would be no restoring torque acting on the subject during the analysis window. The average interaction torque during this period was 0.005·N m. Subjects were encouraged to explore the range of motion before proceeding to the target-matching tasks. Following each trial, the footplate was reset to the starting position to maintain consistency between trials. Similarly to the torque protocol, subjects had five seconds to maintain the position target, and the middle three seconds were used for analysis. The average change in position throughout the analysis window was 0.03°. Following the completion of each trial the mean value was displayed on the plot. As with stiffness and torque tasks, the protocol was repeated for each of the three position targets.

### Statistics and Comparisons

Our goal was to compare the accuracy and precision of joint stiffness regulation to that of joint position and joint torque regulation. This was accomplished by comparing the inter-subject average percent error for all tasks. Percent error was chosen to normalize and compare accuracy and precision across different domains, which eliminated comparisons with different units. Statistical testing consisted of an analysis of variance (ANOVA) with target level (20%, 40%) and domain (stiffness, torque, position) treated as fixed factors, and subject treated as a random factor. Our model also included the interaction between domain and target magnitude. Post-hoc comparisons were performed using Bonferroni corrections, and the significance level of all tasks was set *a priori* to *α* = 0.05. F-scores were presented as *F*_*a*,*b*_ where *a* was the degrees of freedom between groups and *b* was the degrees of freedom within groups. To determine the appropriate number of subjects, a power analysis was completed. Stiffness had the largest variability when compared to torque and position data as reported by Deshpande *et al*.^29^ and our pilot work (Supplementary Tables 4–6), hence it was used to determine the number of subjects. To detect a 10% change in error magnitude, 14 subjects were needed for a statistical power (1-*β*) of 0.8. We chose to detect a change of 10% because it was approximately the average difference in target matching error between stiffness targets in our pilot study.

## Supplementary information

Supplementary tables.
