# Supplementary material for: Neuromotor Regulation of Ankle Stiffness is Comparable to Regulation of Joint Position and Torque at Moderate Levels
Source: Sci Rep. 2020 Jun 25;10:10383. doi: 10.1038/s41598-020-67135-x (PMC7316766; doi:10.1038/s41598-020-67135-x)
Supplement: Supplementary file 1 — Supplementary tables. [file 41598_2020_67135_MOESM1_ESM.pdf]

Subject	20% Stiffness Target	Mean	Std.	40% Stiffness Target	Mean	Std.	60% Stiffness Target	Mean	Std.
1	35.3	40.5	7.6	47.7	48.5	4.37	60.1	60.0	6.3
2	30.5	26.8	3.0	44.1	44.4	13.25	57.7	48.4	11.4
3	30.3	21.5	8.0	44.0	32.0	20.04	57.6	35.7	16.8
4	39.4	39.3	5.8	50.8	52.0	12.22	62.2	59.2	8.5
5	34.9	45.1	11.5	47.4	60.5	16.57	60.0	55.4	15.5
6	28.2	31.7	9.4	42.4	45.9	15.22	56.6	52.3	8.3
7	30.7	30.0	4.0	44.3	41.1	5.87	57.9	51.4	7.0
8	27.4	27.8	4.2	41.8	40.6	7.45	56.2	58.6	9.0
9	26.0	26.3	4.0	40.8	41.3	5.55	55.5	51.4	8.0
10	28.9	23.3	8.0	42.9	23.6	14.1	56.9	22.8	6.9
11	27.3	29.5	9.0	41.8	34.0	11.02	56.2	44.1	17.2
12	38.2	38.9	6.9	49.9	49.3	12.91	61.6	61.4	7.9
13	27.4	31.3	4.2	41.8	41.7	5.64	56.2	54.0	6.3
14	44.5	41.3	13.4	54.7	51.0	11.82	64.8	66.9	10.0

Supplementary Table 1: Stiffness Data (Nm/rad)

Subject	20% Plantar Flexion MVC Target	Mean	Std.	40% Plantar Flexion MVC Target	Mean	Std.	20% Dorsiflexion MVC Target	Mean	Std.
1	-12.9	-14.0	1.5	-25.8	-26.7	2.2	10.8	14.9	6.1
2	-12.5	-15.2	2.1	-24.9	-20.4	1.4	11.5	13.5	1.9
3	-4.6	-8.0	2.1	-9.1	-11.0	1.5	4.2	6.3	0.7
4	-7.0	-9.3	1.2	-14.1	-14.6	2.2	6.7	5.8	0.9
5	-15.4	-16.8	1.8	-30.7	-28.2	3.2	9.5	13.8	1.7
6	-11.1	-11.8	1.9	-22.2	-21.2	3.2	5.7	5.6	0.7
7	-7.8	-6.5	1.2	-15.5	-15.8	1.6	10.2	10.3	1.9
8	-5.3	-7.3	0.7	-10.6	-11.7	1.1	6.7	10.2	5.2
9	-6.7	-5.4	1.1	-13.4	-13.1	0.7	4.9	5.7	1.3
10	-8.2	-7.9	1.2	-16.5	-11.2	1.8	6.6	5.2	1.6
11	-9.0	-9.7	1.7	-17.9	-19.8	3.8	6.4	7.1	2.1
12	-10.5	-10.8	1.2	-21.0	-21.1	2.5	10.2	10.7	0.8
13	-4.4	-6.0	0.9	-8.9	-10.5	1.0	4.0	6.1	1.6
14	-10.1	-12.7	2.7	-20.3	-18.2	3.6	8.5	8.5	1.2

Supplementary Table 2: Torque Data (Nm)

Subject	20% Plantar Flexion Excursion Angle Target	Mean	Std.	40% Plantar Flexion Excursion Angle Target	Mean	Std.	20% Dorsiflexion Excursion Angle Target	Mean	Std.
1	-4.4	-4.5	1.5	-10.8	-10.8	0.8	5.7	5.3	1.1
2	-4.3	-5.6	1.2	-10.8	-5.4	3.3	5.8	4.0	2.3
3	-4.3	-2.9	1.0	-10.8	-10.0	2.5	5.8	3.8	1.5
4	-3.6	-3.6	1.5	-10.2	-11.4	0.9	6.5	6.8	1.1
5	-4.3	-3.9	1.4	-10.8	-11.2	1.1	5.8	6.3	1.0
6	-4.3	-4.5	1.0	-10.8	-8.4	3.3	5.8	5.5	1.4
7	-3.9	-4.1	0.8	-10.4	-10.5	1.3	6.2	6.3	0.8
8	-4.4	-4.6	0.6	-10.8	-10.4	1.0	5.8	5.7	2.3
9	-4.4	-4.6	0.5	-10.8	-7.5	4.3	5.7	5.1	0.8
10	-4.4	-4.2	1.7	-10.8	-9.3	2.6	5.7	6.5	1.9
11	-4.4	-5.1	1.5	-10.8	-10.6	2.8	5.7	6.4	1.4
12	-4.4	-4.8	0.8	-10.8	-9.4	2.5	5.7	6.2	1.1
13	-4.6	-4.0	1.1	-11.1	-11.0	1.3	5.5	5.7	0.7
14	-4.5	-5.8	2.7	-10.9	-11.3	1.7	5.7	5.8	1.9

Supplementary Table 3: Position Data (deg)

Subject	20% Stiffness Target	Mean	Std.	40% Stiffness Target	Mean	Std.	60% Stiffness Target	Mean	Std.
1	22.0	29.3	4.0	43.9	44.2	9.7	65.6	62.9	18.4
2	26.4	26.8	9.2	37.7	37.8	9.7	48.9	50.9	7.4
3	35.4	38.7	6.1	56.2	58.2	7.7	77.0	82.5	16.3
4	40.2	50.1	10.2	54.2	56.0	7.2	68.2	67.4	12.3
5	44.5	61.3	19.7	63.1	72.0	12.7	81.7	82.8	7.7
6	46.7	46.6	8.9	64.5	60.2	8.6	82.2	76.9	14.8
7	28.5	35.7	19.0	42.7	45.4	13.3	56.9	62.6	13.2
8	13.4	21.0	5.8	16.1	20.2	24.7	18.7	45.1	10.1
9	24.8	21.0	7.2	35.4	43.6	22.9	46.1	51.7	18.8

Supplementary Table 4: Pilot Stiffness Data (Nm/rad)

Subject	20% Plantar Flexion MVC Target	Mean	Std.	40% Plantar Flexion MVC Target	Mean	Std.	20% Dorsiflexion MVC Target	Mean	Std.
1	-26.2	-23.8	1.4	-52.4	-46.7	3.0	8.4	8.5	3.1
2	-21.4	-20.3	2.4	-42.8	-40.5	5.6	5.0	5.9	1.1
3	-21.4	-19.6	4.3	-42.8	-40.4	5.6	5.0	5.5	0.7
4	-26.2	-26.2	2.3	-52.4	-47.6	9.0	8.4	9.7	2.7
5	-26.2	-25.1	3.5	-52.4	-48.9	4.6	8.4	8.9	0.7
6	-26.2	-28.1	5.8	-52.4	-42.0	16.0	8.4	10.8	3.3
7	-21.4	-19.8	1.6	-42.8	-38.4	5.4	5.0	6.1	1.5
8	-21.4	-21.1	2.5	-42.8	-22.7	2.0	5.0	5.0	1.5
9	-26.2	-27.7	2.9	-52.4	-49.5	7.8	8.4	10.9	3.1

Supplementary Table 5: Pilot Torque Data (Nm)

Subject	20% Plantar Flexion Excursion Angle Target	Mean	Std.	40% Plantar Flexion Excursion Angle Target	Mean	Std.	20% Dorsiflexion Excursion Angle Target	Mean	Std.
1	-4.2	-4.2	0.7	-10.4	-10.0	2.3	4.8	5.1	1.5
2	-4.2	-4.4	1.0	-10.4	-9.8	1.0	4.8	4.3	1.1
3	-4.2	-4.3	1.7	-10.4	-10.0	1.7	4.8	5.5	1.4
4	-4.3	-4.4	0.9	-10.4	-10.9	1.5	5.7	5.1	1.3
5	-4.4	-4.5	0.8	-10.8	-10.4	1.0	5.7	5.1	1.1
6	-4.4	-4.6	1.1	-10.8	-11.5	1.1	5.7	5.4	0.8
7	-4.4	-4.5	1.6	-10.8	-10.7	2.2	5.6	5.4	0.7
8	-4.4	-4.5	1.5	-10.7	-10.6	0.9	5.7	4.8	2.4
9	-4.4	-4.9	0.5	-10.8	-10.4	2.3	5.7	5.6	1.5

Supplementary Table 6: Pilot Position Data (deg)
